# Expectations of Polish undergraduate medical students for medical humanities classes: a survey-based pilot study

**DOI:** 10.1186/s12909-023-04771-7

**Published:** 2023-10-18

**Authors:** Marta Makowska, Joanna Dec-Pietrowska, Agnieszka J. Szczepek

**Affiliations:** 1 Department of Economic Psychology, Kozminski University, Jagiellońska 57, Warszawa, 03-301 Poland; 2 Faculty of Medicine and Health Sciences, University of Zielona Góra, Zielona Góra, 65-046 Poland; 3 Department of Otorhinolaryngology, Head and Neck Surgery, Charité-Universitätsmedizin Berlin, Corporate Member of Freie Universität Berlin and Humboldt-Universität zu Berlin, 10117 Berlin, Germany

**Keywords:** Medical humanities, Medical education, Medical schools, Medical students’s perception, University menagment, Quality of education, Poland

## Abstract

**Background:**

Medical schools teach Medical Humanities (MH) to provide students with knowledge about the human experience related to health, illness, disease, medicine, and healthcare. Due to the previously observed negative opinions about MH courses, we examined the expectations of medical students in Poland toward humanities subjects.

**Methods:**

We conducted a voluntary, anonymous electronic survey in one medical school (single-center study) and collected data from 166 medical students. The results were analyzed by comparing continuous and categorical variables between groups (gender, year of study, previous participation in MH classes).

**Results:**

The students expected to learn how to communicate with patients and their families, especially about difficult topics. They also expected the classes to be active, stress-free, and without passing grades. The preferred MH teacher was a physician, although choosing a psychologist or other qualified person as an MH teacher was also popular. Previous participants in MH courses were more likely to expect such a course to be compulsory than those who had yet to attend it.

**Conclusion:**

Considering the students’ expectations when designing MH classes could increase students’ satisfaction with MH courses.

**Supplementary Information:**

The online version contains supplementary material available at 10.1186/s12909-023-04771-7.

## Introduction

Medical humanities (MH) is an interdisciplinary specialty concerned with the human experiences of health, disease, illness, medicine, and health care [1]. MH aims to achieve a bilateral understanding between the patient and physician based on the patient’s feelings and experiences and professional medical knowledge. Several medical schools worldwide teach MH courses, but the global outcomes remain to be determined [2, 3]. However, some medical schools reported a positive influence of MH classes on students’ empathy [4, 5] and communication and interpersonal skills [6].

Undergraduate education for future physicians in Poland is available in 22 state-owned or private medical schools [7]. Poland has no pre-med schools, and the students are admitted after high school into a six-year program controlled by the Ministry of Science and Higher Education and the Ministry of Health [8]. Many Polish medical schools have incorporated MH courses into their curricula; however, there are significant differences between universities regarding the form of classes (e.g., lectures, workshops, seminars), the total number of hours dedicated to MH, the content of classes, years of study, and the form of final exams [7].

Our recent qualitative study identified students’ opinions about MH courses and concluded that many were dissatisfied with the content, form, duration, and MH instructors [9]. These opinions were expressed by individuals studying at one of three Polish medical schools in Gdansk, Warsaw, and Kraków, suggesting widespread student dissatisfaction.

The present study explored medical students’ expectations of MH classes to determine what and how they would wish to study. The current investigation was a pilot study in preparation for a larger one involving many medical schools. Exploring students’ expectations could enhance the curricula of medical schools and improve students’ attitudes toward MH.

## Materials and methods

The study was conducted using an online survey posted on the intranet of Collegium Medicum in Zielona Góra, Poland, and accessible between February 1st and March 31st, 2022. Ethics approval was obtained from the Bioethics Committee of the Medical College of the University of Zielona Góra, (approval number RCM-CM-KBUZ.031.11.2023). The link to the questionnaire posted on https://elearning.cm.uz.zgora.pl/ was sent to students by a system administrator. No incentives (e.g., in the form of remuneration) were offered for participation. The system administrator sent four reminders after the initial invitation. Students were informed that participation in the survey is voluntary and anonymous, and its results will be used for scientific purposes. Completing the questionnaire took students, on average, 10 min. The study sample was targeted (medical students of the Collegium Medicum University of Zielona Góra), and the respondents were self-selected. After receiving an e-mail with a link, students independently decided whether to complete the survey.

The study’s authors (J.D-P. and A.J.S) created a survey of 15 questions: five demographic questions about the age, sex, year of study, participation in MH classes, and the year of study when the MH classes were taken. The rest of the questionnaire contained ten main questions, including two open ones (see Supplementary Information). Apart from the questions regarding age, sex, and the year of study, answering other questions was optional and could be omitted. For this article, the most interesting results were chosen.

The survey was addressed to 595 students. Filled questionnaires were returned by 172 students, resulting in a response rate of 28.9%. During the analysis, a decision was made to exclude six filled questionnaires based on students’ answers indicating their participation in MH classes in years higher than the year of study declared. Although such a scenario is possible when the students studied in other faculties before studying medicine, such questionnaires as being suspected of “automatic” completion (without reading) were removed. Ultimately, 166 questionnaires were included in the analysis.

The results were analyzed using the software IBM SPSS Statistics 28. Open questions were coded twice, at a time interval, by one of the persons (MM). Because the data were not normally distributed, non-parametric statistical tests such as Pearson’s chi-square, Mann-Whitney U, and Kruskal-Wallis H tests were used.

## Results

### Sample description

The study sample comprised 66.3% of women and 33.7% of men. The age of the students ranged from 18 to 31 years. More than half of the students (69.3%) studied in the first or second year, whereas 30.7% were more advanced (3rd to 6th year). One-third of the students (31.6%) never took MH courses before the survey.

### Student expectations

Most students (89.8%) expected to be prepared for difficult conversations with patients and their families/relatives by taking MH classes (Table 1). The second highest (81.3%) expectation was being prepared for general contact with patients. The third expectation of 74.1% of students was the desire to communicate better with patients. These three statements gained the most support among both women and men. However, all statements included in Table 1 were selected more frequently by women. For three statements, there was a statistically significant gender difference. Using Pearson’s chi-square test, we determined that female students had agreed with the statement that classes should prepare them for difficult conversations more often (χ2 (1) 5.3 p = 0.021). The same was true for statements: “The MH course should prepare me to deal with patients during mandatory internships” (χ 2 (1) 7.0 p = 0.008) and “The MH course should prepare me to deal with stress and burnout” (χ2 (1) 4.6 p = 0.031). No statistically significant differences were found concerning other statements; however, the results presented in Table 1 show that women agreed with each of the statements more often.

**Table 1:** Students’ expectations regarding the goals of MH courses

Statement	Total (%) *N =* 166	Gender – confirmative statements% (observed counts/expected counts)		Pearson’s Chi-square test
		female	Male	
The MH course should prepare me for difficult conversations with patients and their families/relatives	89.8%	93.6% (103/98.7)	82.1% (46/50.3)	χ2 (1) 5.3 **p = 0.021**
The MH course should prepare me to interact with patients in general	81.3%	82.7% (91/89.5)	78.6% (44/45.5)	χ2 (1) 0.4 p = 0.516
The MH course should prepare me to communicate better with patients	74.1%	74.5% (82/81.5)	73.2% (41/41.5)	χ2 (1) 0.03 p = 0.853
The MH course should prepare me to deal with stress and burnout	68.1%	73.6% (81/74.9)	57.1% (32/38.1)	χ2 (1) 4.6 **p = 0.31**
The MH course should prepare me to interact with colleagues and medical staff	65.1%	68.2% (75/71.6)	58.9% (33/36.4)	χ2 (1) 1.4 p = 0.237
MH course should prepare me to deal with conflicts	65.1%	66.4% (73/71.6)	62.5% (35/36.4)	χ2 (1) 0.2 p = 0.622
The MH course should prepare me to interact with patients during mandatory internships	63.9%	70.9% (78/70.2)	50.0% (28/35.8)	χ2 (1) 7.0 **p = 0.008**

33% of students agreed with all the statements (Table 1), while only 0.6% (1 person) disagreed with all. Mann-Whitney U test determined that women checked more statements than men (U = 2521.5, p = 0.05). The same test did not reveal statistically significant differences between those who had already participated in MH classes and those who had not (U = 3139.0, p = 0.887). There was, however, a statistically significant difference between lower-year students (years 1–2) and more advanced students (years 3–6) [U = 2264.5, p = 0.016], where the less advanced checked more statements.

Only forty-five students responded to an open elective question about their expectations for MH classes. That low response rate prompted us to skip showing the qualitative analysis of the answers to avoid bias.

### Students’ reservations

More than half of the students (54.2%) agreed with the statement that the MH course **would not prepare** them to cope with stress and professional burnout (Table 2). They also questioned whether such courses could prepare them to deal with conflicts (34.9%). The same percentage believed such a course would not prepare them for difficult conversations with patients, their families, and relatives. Pearson’s chi-square revealed no statistical significance between all statements from Table 2 and the subjects’ gender and prior participation in MH classes. Significant statistical differences were shown when the study year was considered. Students at lower years were less likely to agree with each of these statements. Still, a statistically significant difference appeared only in the case of two “MH course will not prepare me for difficult conversations with patients and their families/relatives” (χ2 (1) 8.3, p = 0.004) and “The MH course will not prepare me for better communication with patients” (χ2 (1) 6.4, p = 0.011).

**Table 2:** Analysis of response frequency regarding students’ concerns about course content

Statement	Total (%) *N =* 166	Year of study - confirmative statements % (observed counts/expected counts)		Pearson’s Chi-square
		1–2 years	3–6 years	
The MH course will not prepare me for dealing with stress and burnout	54.2%	52.2% (60/62.3)	58.8 (30/27.7)	χ2 (1) 0.6 p = 0.428
The MH course will not prepare me to deal with conflicts	34.9%	30.4% (35/40.2)	45.1% (23/17.8)	χ2 (1) 3.3 p = 0.068
The MH course will not prepare me for difficult conversations with patients and their families	34.9%	27.8% (32/40.2)	51.0% (26/17.8)	χ2 (1) 8.3 **p = 0.004**
The MH course will not prepare me to interact with colleagues and medical staff	29.5%	25.2% (29/33.9)	39.2% (20/15.2)	χ2 (1) 3.3 p = 0.068
The MH course will not prepare me for better communication with patients	22.9%	17.4% (20/26.3)	35.3% (18/11.7)	χ2 (1) 6.4 **p = 0.011**
The MH course will not prepare me to interact with patients during mandatory internships	20.5%	17.4% (20/23.6)	27.5% (14/10.4)	χ2 (1) 2.2 p = 0.138
The MH course will not prepare me to interact with patients at all	20.5%	19.1% (22/23.6)	23.5% (12/10.4)	χ2 (1) 0.4 p = 0.517

9% of students agreed with all reservations, while 24.7% disagreed with all of them. Mann-Whitney U test was conducted to determine whether there is a difference in the number of statements that respondents agree with some reservations. There were no statistically significant differences between men and women (U = 2978.5, p = 0.724) and between previous MH participants and non-participants (U = 2968.5, p = 0.469). On the other hand, more advanced students agreed with a significantly greater number of reservations than the first and second-year students (U = 2229.5, p = 0.012).

### Expectations regarding the form, frequency, and level of teaching (year of study) of MH classes

Most respondents (38.2%) expected MH classes to be compulsory, whereas 33.9% said they should have a mixed form (some classes should be compulsory, and some elective). More than a quarter of students stated (27.9%) that MH classes should only be elective. Pearson’s chi-square test demonstrated no statistical relationship between gender/being a junior or senior student and views on compulsory or the elective character of the classes. However, there was a statistically significant difference (χ2 (2) 7.8p = 0.020) between previous MH class participants and non-participants. The distribution analysis of the frequencies (Table 3) shows that students who previously attended MH classes expect that such classes should be compulsory (40.6%) or mixed (38.7%). On the other hand, 40.7% of those who previously did not take MH classes expected elective classes.

**Table 3:** Analysis of compulsory/elective preferences depending on sex, previous participation in MH classes, and the year of study

	Format of classes as % of confirmative answers (observed counts/expected counts)			Pearson’s chi-square
	Compulsory *N* = 63	Elective *N* = 46	Mixed *N* = 56	
Women	35.8% (39/41.6)	29.4% (32/30.4)	34.9% (38/37)	χ2 (2) 0.8 p = 0.664
Men	42.9% (24/21.4)	25.0% (14/15.5)	32.1% (18/19)	
earlier MH participants	40.6% (43/40.5)	20.8% (22/29.6)	38.7% (41/36)	χ2 (2) 7.8 **p = 0.020**
not yet participated in MH	33.9% (20/22.5)	40.7 (24/16.4)	25.4% (15/20)	
1–2 year	36.5% (42/43.9)	27.8% (32/32.1)	35.7% (41/39)	χ2 (2) 0.6 p = 0.740
3–6 year	42.0% (21/19.1)	28.0% (14/13.9)	30.0% (15/17)	

*N* = 166

There was a statistically significant difference, as measured by the Kruskal-Wallis test, between the number of statements related to student’s expectations for the skills learned (see Table 1) and the expected class form (compulsory, elective, or mixed) and showed a statistically significant difference (H (2) = 8.4, p = 0.015). Those who chose the mixed form had the highest expectations (mean rank 94.1), followed by those who favored compulsory (84.5), and finally, those who preferred the elective form of classes (67.4). No statistically significant difference existed in the number of statements related to students’ reservations (Table 2) (H(2) = 0.9, p = 0.635).

In addition, an open question was posed (“Please briefly justify your preferred type of classes (compulsory or elective)”). Eighty-four respondents answered this question: 45 indicated that classes should be compulsory, 20 - elective, and 19 – mixed (Table 4). The students expecting compulsory courses motivated their choice (82.2%) by gaining skills necessary in medical practice (e.g., communication/dealing with difficult situations); 8.9% stated that MH classes provide mandatory knowledge; another 8.9% believed that when the MH classes were not mandatory, they would be ignored. Among those who indicated that MH classes should be elective, the most common explanation (85%) was that only students interested in the subject should attend them, as they benefit the most. Of the latter responders, 40.0% had reservations about MH classes (e.g., that they take up time and do not contribute much). Most students who expected mixed classes (84.2%) justified their answer by MH basic knowledge being mandatory. These students believed that if someone wants to broaden their knowledge, they should be able to do so during elective courses. Equal parts of the latter group (21.1%) noticed that either MH classes gave practical skills or criticized them. A small percentage (5.3%) of students expressed their opinion about the knowledge of HM being mandatory, without adding an explanation for the elective option.

**Table 4:** Analysis of open question about preffered type of class

	Expected form of classes % (n)			Total	Example
	compulsory *N* = 45	elective *N* = 20	mixed *N* = 19		
MH classes provide skills necessary in practice (e.g., communication/difficult situations)	82.2% (37)	10.0% (2)	21.1% (4)	43	“Many physicians have communication problems; the patient’s health deteriorates because of their behavior and statements; they should have better-developed skills under the guidance of specialists.”
Criticism of classes (they do not add anything; they take too much time; the topics overlap; not everyone can learn empathy; they have bad form; one has to agree with the teacher).	4.4% (2)	40.0% (8)	21.1% (4)	14	“The most important is substantial knowledge, and it cannot be sacrificed for the MH”; “(…) if someone has not “learned empathy” by this point in their life, no course will change that (…)”
Interested people should attend the MH classes - they benefit the most	2.2% (1)	85.0% (15)	10.5% (2)	twenty	“If someone does not want to participate in the classes, they cannot benefit from them and will show up only for the attendance, which makes no sense.”
MH is a mandatory knowledge	8.9% (4)	0% (0)	5.3% (1)	5	“this is a must-know”
If the MH classes are not mandatory, they will be ignored.	8.9% (4)	0% (0)	0% (0)	4	“I think if such classes were elective, students could downplay them and ignore them in favor of choosing other classes, so it would be better if they were compulsory.”
Mandatory + elective	0% (0)	0% (0)	84.2% (16)	16	“All students should attend a course on humanizing medicine/communication. In addition, there should be an elective on this subject in higher years for students who want to improve these skills.“
Other	4.4% (2)	5.0% (1)	0% (0)	3	“[the classes should be] mandatory due to foreign guidelines of teaching medical students.“

Note: Percentages do not add up to 100% because responses were coded into multiple categories

### Expectations concerning the way of crediting MH classes

**Fig. 1:**
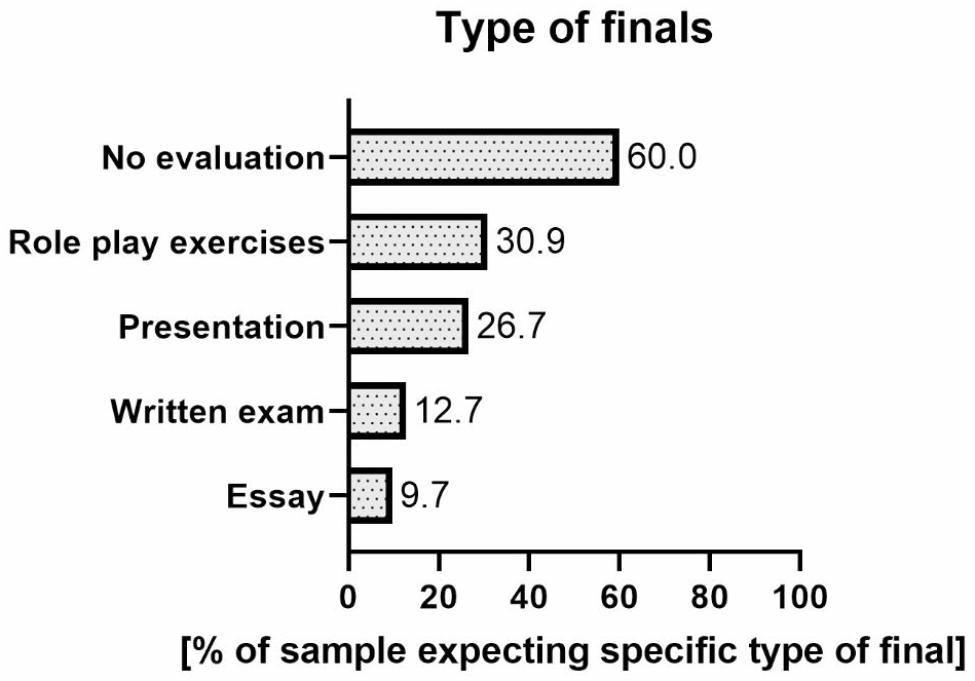
Expectations concerning the form of finals (N = 166). Note: Percentages do not add up to 100% because respondents could choose more than one answer

Most survey participants expected no evaluation and disagreed with having other forms of finals (Fig. 1). There was a significant difference between those declaring the way of passing and the form of MH classes they chose (χ2 (2) 14.0 p < 0.001). More students who preferred the elective class wanted no grading of their MH knowledge than expected.

Almost one-third of students (30.9%) believed that role-play exercises are an excellent way to pass this class. There was a statistically significant difference between the form of classes preferred by students and the type of crediting (χ2 (2) 10.0 p = 0.007). Students who chose the elective class preferred this way of crediting less frequently than expected.

About 26% of students expected their MH knowledge to be assessed during a presentation. There was also a statistically significant difference between the form of classes and the credit (χ2 (2) 11.3 p = 0.003). Fewer students than expected, preferring the elective classes, chose this form of credit (for details see Table 5).

### Expectations regarding the format of MH classes

**Fig. 2:**
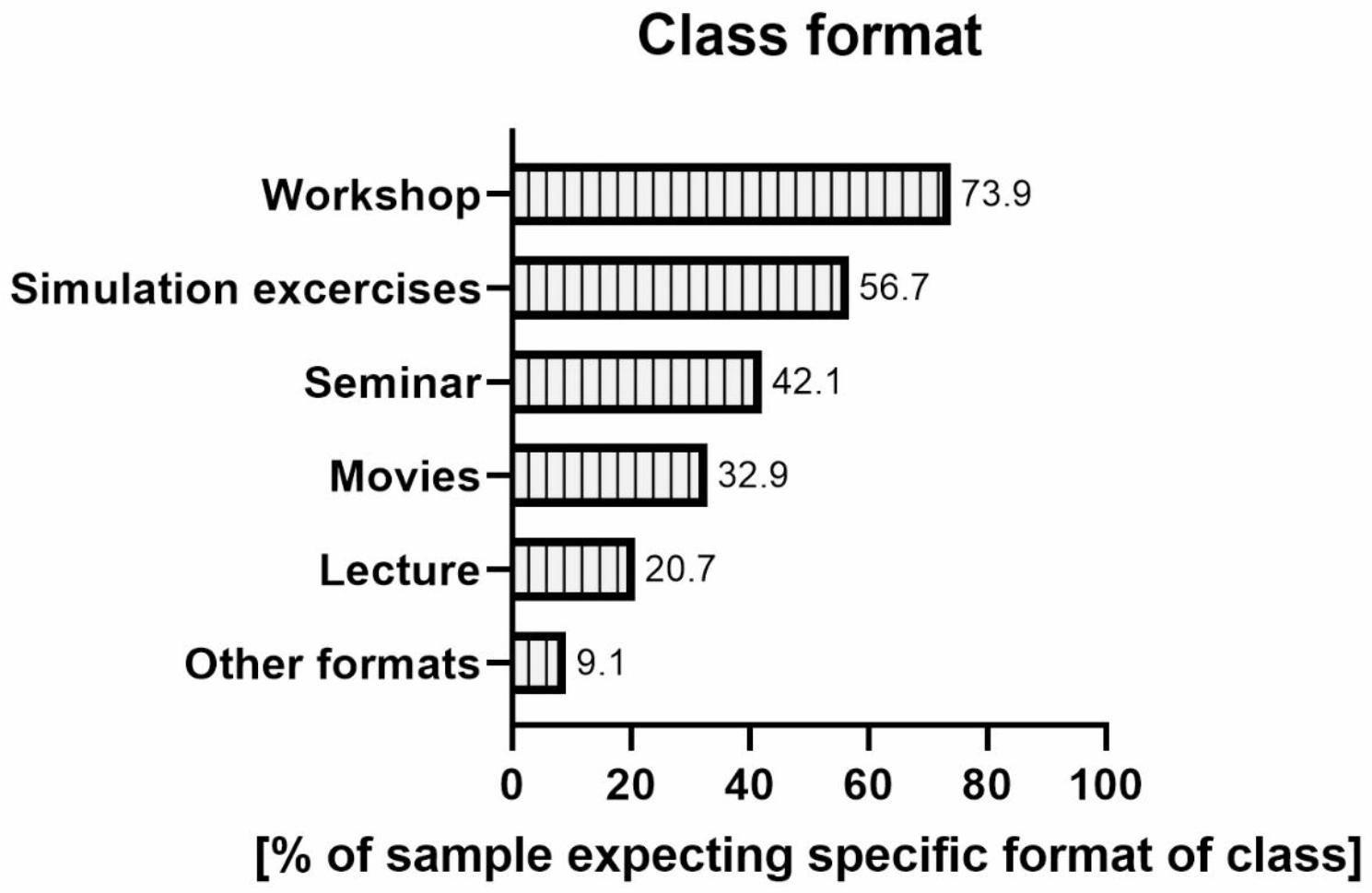
Expectations concerning the format of HM classes (N = 166). Note: Percentages do not add up to 100% because respondents could choose more than one answer

The greatest number of students expected MH classes to be organized as workshops (73.9%) (Fig. 2). There was a statistical relationship between this view and the preferred form of classes (χ2 (2) 11.5 p = 0.003). Among those who chose the elective form, more students than expected did not opt for a workshop. According to students, simulation (56.7%) was the next most desirable method of carrying out these classes, followed by a seminar (42.1%). For details see Table 5.

**Table 5:** Form of finals and class format preferences stratified based on students’ preferred form of class

	Expected form of classes - % (observed counts/expected counts)			Pearson’s
	compulsory	elective	mixed	Chi-square
Form of finals				
Written exam				
No	82.5% (52/55)	95.7% (44/40.1)	85.7% (48/48.9)	χ2 (2) 4.3
Yes	17.5% (11/8)	4.3% (2/5.9)	14.3% (8/7.1)	p = 0.116
Essay				
No	85.7% (54/56.9)	97.8% (45/42.5)	89.3% (50/50.6)	χ2 (2) 4.5
Yes	14.3% (9/6.1)	2.2% (1/4.5)	10.7% (6/5.4)	p = 0.103
Presentation				
No	69.8% (44/46.2)	91.3% (42/33.7)	62.5% (35/41.1)	χ2 (2) 11.3
Yes	30.2% (19/16.8)	8.7% (4/12.3)	37.5% (21/14.9)	**p = 0.003**
Role play exercises				
No	65.1% (41/43.5)	87.0% (40/31.8)	58.9% (33/38.7)	χ2 (2) 10.0
Yes	34.9% (22/19.5)	13.0% (6/14.2)	41.1% (23/17.3)	**p = 0.007**
disagree	46.0% (29/25.2)	17.4% (8/18.4)	51.8% (29/22.4)	χ2 (2) 14.0
agree	54.0% (34/37.8)	82.6% (38/27.6)	48.2%(27/33.6)	**p < 0.001**
Class format				
Workshop				
No	15.9%(10/16.5)	44.4% (20/11.8)	23.2% (13/14.7)	χ2 (2) 11.5
Yes	84.1% (53/46.5)	55.6% (25/33.2)	76.8% (43/41.3)	**p = 0.003**
Simulation excercises				
No	44.4% (28/27.3)	46.7% (21/19.5)	39.3% (22/24.2)	χ2 (2) 0.6
Yes	55.6% (35/35.7)	53.3% (24/25.5)	60.7% (34/31.8)	p = 0.738
Seminar				
No	54.0% (34/36.5)	62.2% (28/26.1)	58.9% (33/32.4)	χ2 (2) 0.8
Yes	46.0% (29/26.5)	37.8% (17/18.9)	41.1% (23/23.6)	p = 0.681
Lecture				
No	77.8% (49/49.9)	75.6% (34/35.7)	83.9% (47/44.4)	χ2 (2) 1.2
Yes	22.2% (14/13.1)	24.4% (11/9.3)	16.1% (9/11.6)	p = 0.548
Movies				
No	63.5% (40/42.3)	73.3% (33/30.2)	66.1% (37/37.6)	χ2 (2) 1.2
Yes	36.5% (23/20.7)	26.7% (12/14.8)	33.9% (19/18.4)	p = 0.552
Other forms				
No	90.5% (57/57.2)	88.9% (40/40.9)	92.9% (52/50.9)	χ2 (2) 0.5
Yes	9.5% (6/5.8)	11.1% (5/4.1)	7.1% (4/5.1)	p = 0.783

Note: A student could choose multiple forms of credit. Therefore, the sum of affirmative answers is not 100%

### Expectations regarding the MH instructors

**Fig. 3:**
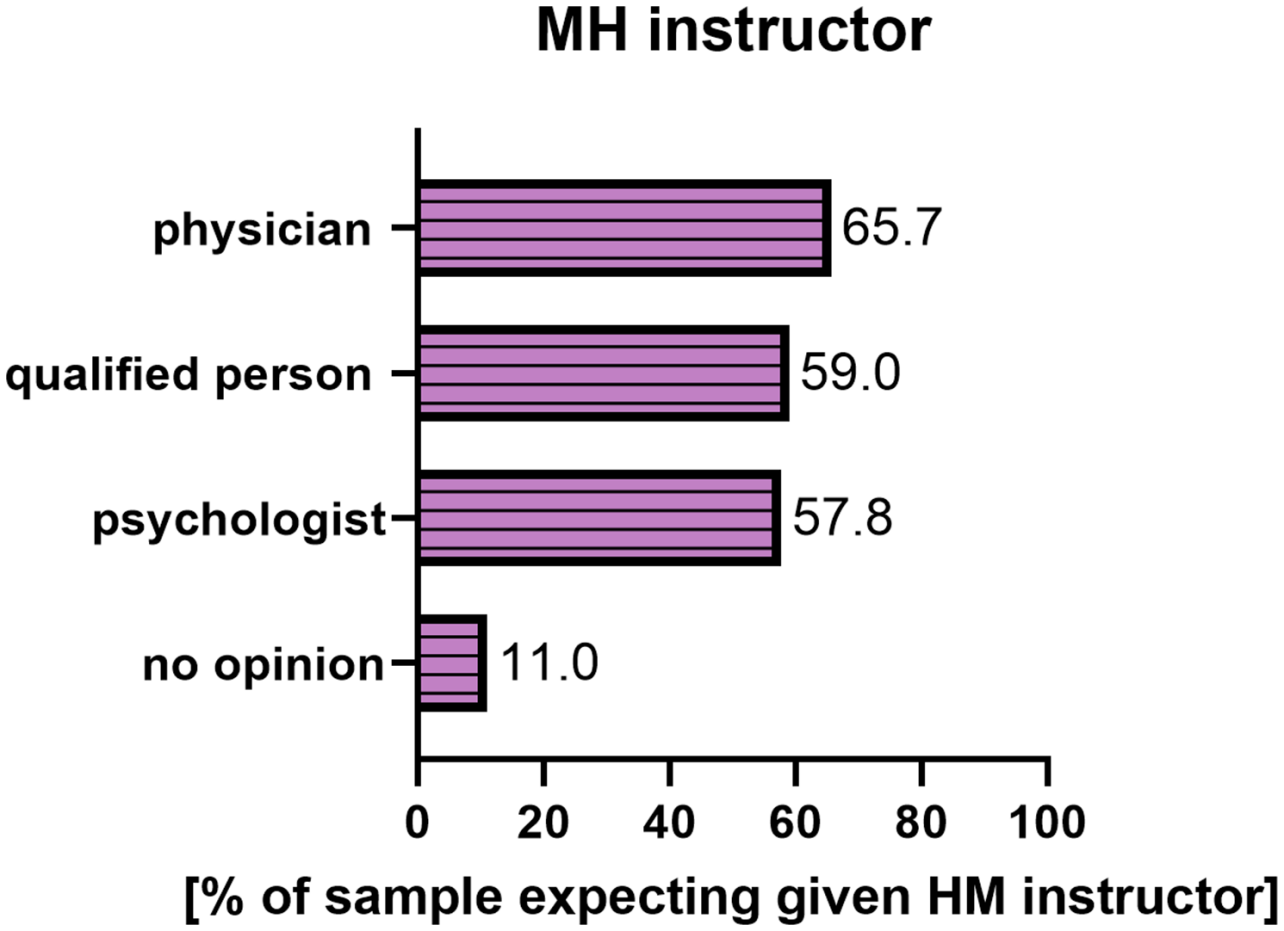
MH instructor preference among medical students at the University of Zielona Góra (N = 166). Note: Percentages do not add up to 100% because respondents were asked about each instructor separately

Most students (65.7%) expected a physician as an MH instructor, 59.0% indicated that it could be another qualified person and 57.8% that it could be a psychologist. Almost one in ten students (11%) had no opinion on who should conduct MH classes (Fig. 3**).** Responders who chose the elective classes were the biggest supporters of having a physician as an instructor (73.9%). Of note, Pearson’s chi-square has not determined the statistical significance between the choice of a physician and the preferred form of classes (χ2 (2) 3.4 p = 0.184). Statistically significant differences appeared in the case of choosing a psychologist and the form of classes (χ2 (2) 6.5 p = 0.040). Those who preferred mandatory courses were most likely (69.8%) to agree that a psychologist could teach such courses, while those who preferred optional courses were least likely (47.8%) to agree. There was also a statistically significant difference between the choice of another qualified person and the form of the course (χ2 (2) 6.9 p = 0.032). Again, it was most often allowed by those who preferred mandatory classes (68.3%) and least often by those who chose optional classes (43.5%).

## Discussion

To the best of our knowledge, our study is currently the only one that analyzes the expectations of Polish medical students regarding MH classes. A similar study was performed in the UK by Petrou et al. at the Imperial College School of Medicine of London and focused on students’ perspectives regarding MH [10]. However, that study was designed not to assess students’ expectations, but “to elicit demographics, engagement, interest and perspective on curricular positioning, and to explore how students ranked the qualities of a doctor”. Nevertheless, some of the results obtained by Petrou et al. will be compared to ours, and the similarities and differences will be discussed.

Our research determined two main expectations of medical students of MH: (1) they expected that the MH classes will prepare them for interactions with patients, and (2) they also expected to be prepared during MH for difficult conversations with patients and their families.

We determined significant sex-dependent differences regarding the expectations for MH (Table 1). Women chose significantly more statements verbalizing the expectations, indicating higher expectations than men. A feasible explanation could be that men and women are expected to fulfill gender-appropriate social roles, which differ. Women are assigned to mastering skills such as sensitivity and communication [11] because they are often the caregivers of children and older adults. Hence, women may have a greater interest in learning MH. Additionally, women have better non-verbal communication skills than men [11]. Some studies show that women are more empathic among medical students than men [12–16]. It is possible that already possessing empathic and communication skills encourages their further development. One study showed that female medical students are less confident and more anxious than their male colleagues when first meeting patients, so perhaps MH classes help women overcome this lack of self-confidence [17]. Indeed, these higher expectations of women towards MH classes need further exploration in future research. The study by Petrou et al. has not identified gender-dependent differences [10], but this aspect of expectations number was not in focus there.

Another interesting finding of our study was that less advanced students had more expectations than more advanced students, and more advanced students had more reservations about HM than less advanced students. That could be explained by decreased empathy, causing more significant skepticism of senior students regarding their expectations regarding MH courses. Research indicates that students’ empathy falls during medical studies [13, 16], and some studies find that Western students are more susceptible to loss of empathy than Asians [13]. Another factor possibly explaining our results is cynicism among medical students, which increases during the medical study period [18]. Older students are considered cynical as they focus mainly on the physical aspects of the disease because medical studies especially test this side of their skills while ignoring the social and psychological factors. Thus, the desire to pass exams can inhibit the need to explore a humanistic side of medicine, and even the belief that a student will learn specific MH skills decreases, as seen in the present study. This doubt may result from the fact that the goals of MH, such as fostering empathy, learning critical thinking, and thoughtfulness, may seem simply unachievable to some [19]. In agreement with that, some students in the survey believe that empathy cannot be learned, and this opinion is supported by published research [20]. In contrast, studies have shown that classes in social sciences, literature, poetry, and history can influence and develop empathy [19]. In addition, some other studies describe the success of courses enhancing empathy among undergraduate medical students [21], and therefore, it would be advisable for similar studies to be conducted in Poland.

The presented study demonstrates that students who previously participated in the MH courses most often expect them to be compulsory. On the other hand, those who had not taken an MH course expected it to be an elective. This observation suggests that the content of the MH course is more relevant to students who have taken it than to students who have not. The study by Petrou et al. also showed a dichotomy of opinion regarding the optional or compulsory format of MH courses, but it was not stratified by the advancement of medical studies [10]. Howick et al. (2021) reported that in Canada, 56% of medical schools offer at least one MH course (researchers excluded medical ethics), and 33% of schools held this course as compulsory [22]. In the UK, 73% of schools offered MH, but only 12% considered it mandatory. In the USA, 80% of schools had such a course, and 57% held it as compulsory. A similar study in Italy and Spain determined that all medical schools had at least one MH subject [23]. History and philosophy classes (with bioethics) were most often obligatory in both countries. All 15 Polish medical schools included in the previously published analysis had compulsory MH courses [7], indicating a developing trend in Poland. Still, students and medical school administrators may have trouble appreciating the value of MH courses for future medical practice [19].

Students’ most desired form of classes was using active methods, e.g., workshops or simulations. That is not surprising, as activating forms of courses are more attractive to students than passive ones [24]. It is also an effective form of learning, as evidenced by a systematic review from 2017, indicating that classes for healthcare students conducted with the help of simulation “improve knowledge, skills, and self-confidence” [25]. Another observation we made was that the students who expected to have MH as an elective class chose significantly less frequently an active class format, such as a workshop. A recently published study about students’ satisfaction with a “flipped classroom” teaching method that requires active participation determined that 61% of the medical students were happy with this learning format, whereas, for 24%, it did not matter and induced a negative response in 15% [18], which roughly agrees with a tendency seen in our work.

Most students in the present study (60%) did not expect MH courses to end with an evaluation, similar to what the study of Petrou et al. has shown [10]. Within the group that expected elective MH classes, 82.6% expected no finals, much more than those who expected mandatory (54.0%) or hybrid courses (48.2%). On the one hand, a lack of final tests may prime students to disregard what is happening in class and be reluctant to participate in discussions. On the other hand, the lack of graded finals offers the opportunity to participate in classes that do not require learning by heart, are stress-free, use activating methods, and can give students much satisfaction. Moving away from grades is an increasingly popular concept at various levels of education [26].

The last but essential issue determined by our study was the different expectations of students regarding the MH instructors. The highest proportion of students expected their instructor to be a physician. Shapiro et al. observed and described the trend of having a preferred MH teacher as a physician [27]. This trend was explained by students perceiving non-physicians as “outsiders” who do not understand the clinical realities. At the same time, in our study, we could not determine the statistical significance between the expectations of having a physician, psychologist, or a qualified person teaching the course. However, unlike in other countries, Polish universities do not offer MH undergraduate studies or education for future MH instructors. Therefore, the definition of a “qualified person” as an MH instructor is, in this case, vague, and further study should determine the professional background of MH teachers in Poland.

### Study limitations

The limitation of our study is a small, non-representative sample due to self-selection and the use of only one medical school. Additionally, the students from lower years of study were overrepresented in our sample. These drawbacks could be addressed in a more extensive study involving several medical schools and different sampling methods, allowing a better representation of Polish students.

### Future research

One of the most exciting results was that students who had previously attended MH courses were statistically more likely to believe that such a course should be compulsory than those who had yet to attend it. That can indicate an appreciation of the content of the MH course after taking it. Alternatively, the expectation of MH courses to be compulsory could represent a form of schadenfreude previously found among undergraduate students [28]. Since our sample concerned only one medical school, this theme should be explored in further research in other medical universities, testing how the learning experience influences teaching expectations and using tools such as Freudenfreude and Schadenfreude Test (FAST) to add more explanatory dimensions to the present findings.

### Conclusions

**Fig. 4:**
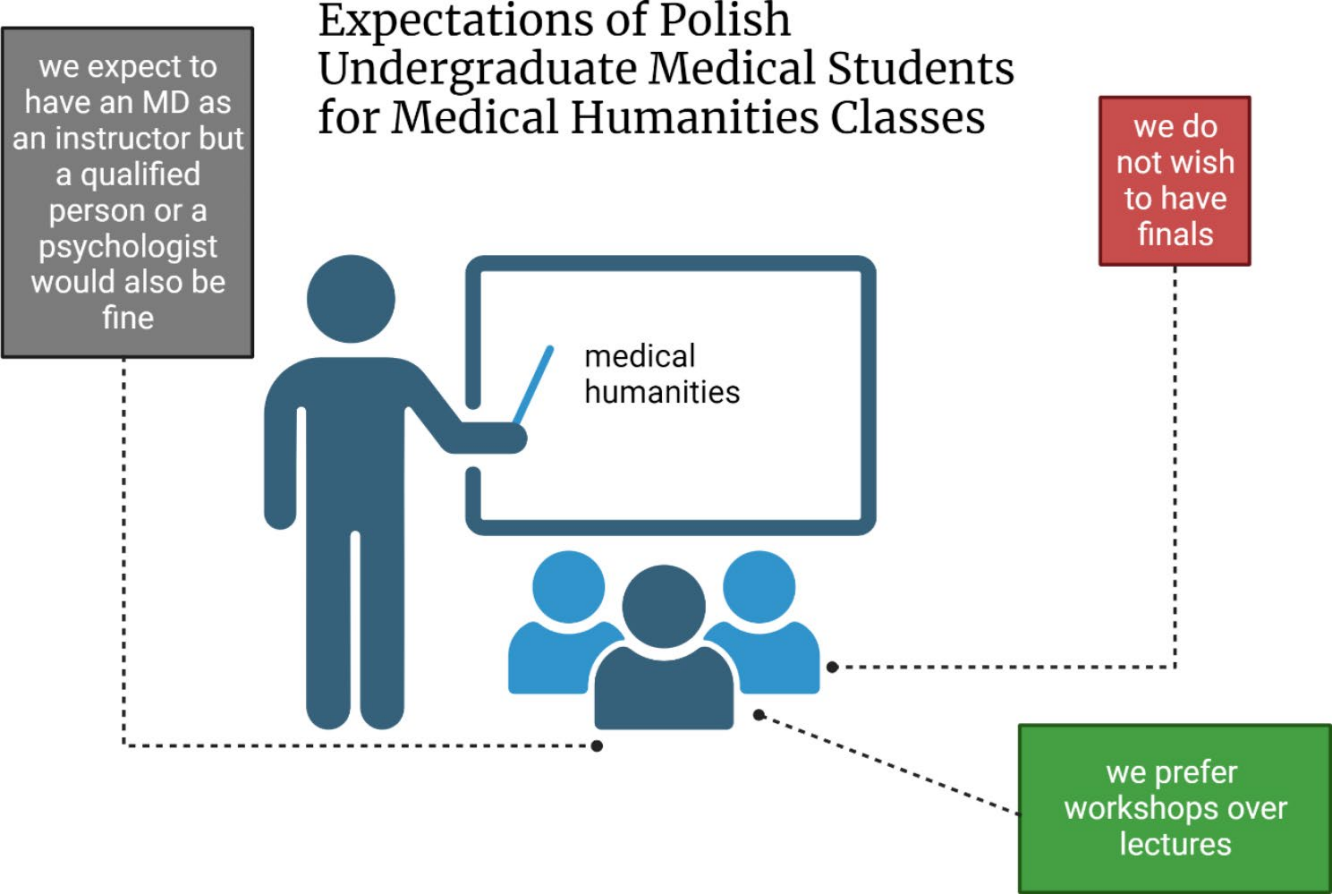
Graphical summary of the main findings. Created with BioRender.com

Most of the medical students who participated in the study stated that they needed practical preparation for interacting with patients.The students expected to learn how to communicate with patients and their families, especially about difficult topics. They also anticipated that classes should be conducted in an active, stress-free format, preferably without passing grades (Fig. 4). The preferred teacher was a physician, although choosing a psychologist or other qualified person as an MH teacher was also popular. Women expected to learn more during MH courses than men. Considering students’ expectations when designing MH classes will likely increase student satisfaction with MH courses.

### Electronic supplementary material

Below is the link to the electronic supplementary material.

Supplementary Material 1

## Data Availability

The datasets used are available from the corresponding author on request.
