# Supplementary material for: Expectations of Polish undergraduate medical students for medical humanities classes: a survey-based pilot study
Source: BMC Med Educ. 2023 Oct 18;23:775. doi: 10.1186/s12909-023-04771-7 (PMC10585903; doi:10.1186/s12909-023-04771-7)
Supplement: Supplementary file 1 — Supplementary Material 1 [file 12909_2023_4771_MOESM1_ESM.docx]

Survey “Students’ expectations from MH courses.”

1. How old are you?

1. Sex

Woman
Man
Diverse

1. Before this survey, have you taken any MH courses?

1. In which year are you studying at the moment?

a . 1st year
b. 2nd year
c. 3rd year
d. 4th year
e. 5th year
f. 6th year

1. If you participated in courses related to MH, in which year?

a . 1st year
b. 2nd year
c. 3rd year
d. 4th year
e. 5th year
f. 6th year

1. Which of the following statements reflects your expectations for MH courses? (Please check all that is important to you)

a . The MH course should prepare me to interact with patients during mandatory internships
b. The MH course should prepare me to interact with patients in general
c. The MH course should prepare me to interact with colleagues and medical staff
d. MH course should prepare me to deal with conflicts
e. The MH course should prepare me for difficult conversations with patients and their families/relatives
f. The MH course should prepare me to communicate better with patients
g. The MH course should prepare me to deal with stress and burnout

1. Which of the following statements reflect your reservations about medical humanization/communication courses/subjects? (please check everything that is important to you)

a . The MH course will not prepare me to interact with patients during mandatory internships
b. The MH course will not prepare me to interact with patients at all
c. The MH course will not prepare me to interact with colleagues and medical staff
d. The MH course will not prepare me to deal with conflicts
e. The MH course will not prepare me for difficult conversations with patients and their families
f. The MH course will not prepare me for better communication with patients
g. The MH course will not prepare me for dealing with stress and burnout

1. In which year should MH classes be organized to be most effective?

a . In 1st year
b. In 2nd year
c. In 3rd year
d. In 4th year
e. In 5th year
f. In 6th year
g. clinical items

1. Should MH classes be:

a . Compulsory
b. Elective
c. Mixed

1. Please briefly justify your preferred type of classes (compulsory or elective)

1. How should the MH knowledge be graded?

a . Written - knowledge test
b. Written work/essay
c. Presentation during the course/class of a selected problem topic
d. Role-playing/describing a scene on a selected problem topic
e. Should not be graded

1. Who should teach MH classes?

a . Physician
b. Psychologist
c. Trained person
d. I have no opinion

1. If you were an academic teacher, what format of classes would you use to teach MH classes?

i. Workshop
II. Simulation with professional actors
iii. Seminars
iv. Lecture
v. Educational films
vi. other formats

1. If you were an academic teacher, what content would you like to convey during MH classes?

iv. Clinical practice-oriented content
ii. Only a few definitions
iii. Many definitions
iv. Much history
v. Minimal history
vi. The use of fine arts in medical practice

1. What additional expectations, which are not covered by this survey, would you have regarding MH classes?

Bottom of Form
